# The current trends and future perspectives of prebiotics research: a review

**DOI:** 10.1007/s13205-012-0044-x

**Published:** 2012-01-28

**Authors:** Seema Patel, Arun Goyal

**Affiliations:** 1 Department of Biotechnology, Lovely Professional University, Jalandhar, 144402 Punjab India; 2 Department of Biotechnology, Indian Institute of Technology Guwahati, Guwahati, 781039 Assam India

**Keywords:** Prebiotics, Oligosaccharides, Anticancer, Colitis prevention, Cholesterol reduction

## Abstract

Prebiotics are non-digestible food ingredients that stimulate the growth of bifidogenic and lactic acid bacteria in the gastro-intestinal tract. Typically, the prebiotics consist of dietary fibers and oligosaccharides. Prebiotics exert a plethora of health-promoting effects, owing to which multi million food and pharma industries have been established. Prebiotics are being implicated in starter culture formulation, gut health maintenance, colitis prevention, cancer inhibition, immunopotentiaton, cholesterol removal, reduction of cardiovascular disease, prevention of obesity and constipation, bacteriocin production, use in fishery, poultry, pig, cattle feed and pet food. Looking at the ever-increasing demand of prebiotics, in this review, recent trends in prebiotic production from new novel sources, from food industrial wastes, prebiotic supplementation in food, commercially available prebiotic agents, prebiotic production by various techniques and future perspectives has been discussed. The critical insight into this hot research area aims to stimulate further ponderance.

## Introduction

Prebiotics are generally defined as non-digestible polysaccharides and oligosaccharides (NDO), which promote the growth of beneficial lactic acid bacteria in the colon and exert antagonism to *Salmonella* sp. or *Escherichia coli*, limiting their proliferation. The term prebiotics was coined by Gibson and Roberfroid (1995). Gibson et al. (2004) elaborated the prebiotics concept by certain criteria viz*.* resistance to gastric acidity, hydrolysis by mammalian enzymes and gastrointestinal absorption; fermentation by intestinal microflora and selective stimulation of the growth, and/or activity of intestinal bacteria associated with health and wellbeing. There exists an array of prebiotics with various origin and chemical properties. Stowell (2007) reviewed the existing prebiotics and classified them based on a set of common criteria. Inulin, fructooligosaccharides (FOS), galactooligosaccharides (GOS), lactulose and polydextose are recognized as the established prebiotics, whereas isomaltooligosaccharides (IMO), xylooligosaccahrides (XOS), and lactitol are categorized as emerging prebiotics. Chicory root inulin-derived (FOS), wheat bran-derived arabinoxylooligosaccharides (AXOS) and xylooligosaccharides (XOS) proved to have huge applications (Sabater-Molina et al. 2009; Femia et al. 2010; Xu et al. 2009). Mannitol, maltodextrin, raffinose, lactulose, and sorbitol are also prebiotics with proven health properties (Yeo and Liong 2010; Vamanu and Vamanu 2010; Mandal et al. 2009). Resistant starch-rich whole grains are considered prebiotic in nature and assumed that their consumption leads to many health benefits. These are not absorbed in small intestine of healthy individuals but later are fermented by natural microflora of the colon to produce short-chain fatty acids (SCHFA) (Vaidya and Sheth 2010). The fermentability of dietary fibers viz. oat β-glucan, flaxseed gum, and fenugreek gum to SCFAs suggest their potential prebiotic application in promoting human health (Lin et al. 2011). Of late, mannan oligosaccharide-rich yeast cell wall material is demonstrated to be a valuable prebiotic.

Due to poor nutrition, tobacco and alcohol consumption, the past few decades have seen alarming increase in morbidity and mortality. With instances of chronic obesity, gastrointestinal disorders, diabetes, coronary diseases, cancers, and degenerative diseases on the rise, growing numbers of consumers are looking up to companies manufacturing prebiotics. Cashing in on the consumer craze for low-carbohydrate high-fiber diet, nutraceutical market is being dominated by a wide range of prebiotic products. First Leaf (FL; composed of blackcurrant extract powder, lactoferrin and lutein) developed by the Four Leaf Japan Co. Ltd, Japan, and Cassis Anthomix 30 (CAM30; blackcurrant extract powder) developed by Just the Berries Ltd, New Zealand) are health supplement ingredients emerging as suitable prebiotic agents. Gavaging rats with CAM30 and FL significantly increased the numbers of bifidobacteria and lactobacilli and decreased the numbers of bacteroides and clostridia. Also it exhibited reduction in the activity of β-glucuronidase and increment in the activity of β-glucosidase. Molan et al. (2010) concluded that these health benefits may render these products good source of prebiotics. After 20 days’ supplementation of wheat germ preparation Viogerm^®^PB1 to human subjects, the coliform population and pH decreased significantly, and the number of lactobacilli and bifidobacteria increased significantly. These results showed that the product Viogerm^®^PB1 possesses a prebiotic effect and has a potential to improve host’s health (Matteuzzi et al. 2004). Glover et al. (2009) evaluated the effect of matured gum arabic (*Acacia (sen) SUPERGUM*^™^) supplementation on systolic blood pressure of normal individuals and diabetic nephropathy patients. The dietary administration of 25 g *SUPERGUM*^™^ daily for 8–12 weeks exerted significant beneficial effect on blood pressure of both groups. *SUPERGUM*^™^ has been produced with structural reproducibility, which shows both in vivo and in vitro therapeutic effect on diabetes mellitus and reduction in systolic blood pressure (Phillips and Phillips 2011).

Pineiro et al. (2008) reviewed in the technical meeting convened by Food and Agriculture Organization of the United Nations (FAO) the beneficial effect of prebiotics on food. A panel of international experts set guidelines, recommended criteria, and formulated a systematic approach for the evaluation of prebiotics for ensuring their safe use.

**Fig. 1:**
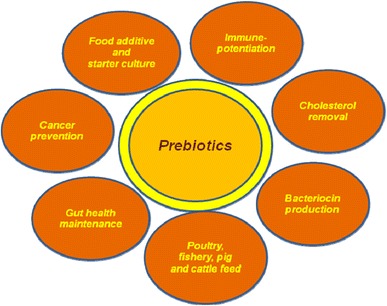
Potential applications of prebiotics

The aim of this review was to summarize the latest findings on prebiotic research, to explore and unravel the new sources of prebiotics, their production techniques, and future directions. The current and envisaged applications of prebiotics are presented in Fig. 1.

## Nutraceutical and pharmaceutical importance of prebiotics

### Food additive and in starter culture

Soluble fibers of inulin influence dough and bread quality (Hager et al. 2011). Inulin is used as a prebiotic to improve the quality of skim milk fermented by pure cultures of *Lactobacillus acidophilus*, *Lactobacillus rhamnosus*, *Lactobacillus bulgaricus* and *Bifidobacterium lactis*, *Streptococcus thermophilus*, or a cocktail containing all them. Inulin supplementation to pure cultures has been evidenced to lower the generation time of *S*. *thermophilus* and *L*. *acidophilus* (Oliveira et al. 2011). Rodrigues et al. (2012) investigated the influence of FOS and inulin (50:50) on the free fatty acid profile of cheese, with special emphasis on the conjugated linoleic acid. Increase of the conjugated linoleic acid content during the ripening time suggests the addition of prebiotics in probiotic cheese manufacture, for better quality product with lower atherogenicity index. Herfel et al. (2011) evaluated the prebiotic effect of polydextrose enrichment in cow milk-based infant formula using 1-day-old piglets for 18 days. Results demonstrated increased ileal lactobacilli population, enhanced propionic and lactic acid concentrations, and decreased pH. Infants suffering from the genetic disorder phenylketonuria (PKU) cannot metabolize phenylalanine in the diet and need infant formula free of this amino acid. MacDonald et al. (2011) investigated the influence of adding a specific mixture of prebiotic oligosaccharides to a protein substitute diet using a single-arm study in nine infants diagnosed with PKU. PKU Anamix Infant, a diet designed for infants with PKU has prebiotic oligosaccharide content of 0.8 g/100 mls, a GOS/FOS blend similar to that in breast milk. It maintained phenylalanine in control, enhanced the levels of *bifidobacteria,* and lowered stool pH. Supplementation of soy milk with FOS and maltodextrin increased the α-galactosidase activity of probiotics viz*. L. acidophilus* FTDC 8033, *L. acidophilus* ATCC 4356, *Lactobacillus casei* ATCC 393, *Bifidobacterium* FTDC 8943, and *Bifidobacterium longum* FTDC 8643, leading to enhanced hydrolysis and utilization of fructose and glucose, promoting their growth (Yeo and Liong 2010).

### Gut health maintenance, colitis and constipation prevention

Prebiotics have a positive influence on the gut-associated lymphoid tissues (GALT). Inulin, FOS, mannooligosaccharides, and arabinogalactans are therapeutic nutritional preparations used for the optimum gut function for favoring the proliferation of normal bacterial flora and impeding the growth of pathogenic organisms. The consumption of prebiotics can modulate immune parameters in GALT, secondary lymphoid tissues and peripheral circulation (Bodera 2008). Necrotizing enterocolitis (NEC) is a major cause of morbidity and mortality in premature infants. Probiotic and prebiotic administration manipulate the intestinal bacterial community, accelerating the growth of commensal bacteria. In vivo experimental result from animal studies and human trials suggest that probiotics decrease the incidence of NEC (Stenger et al. 2010). Prebiotic supplemented formula increase stool colony counts of bifidobacteria and lactobacilli in preterm neonates without adversely affecting weight gain (Srinivasjois et al. 2009). FOS are being increasingly included in food products and infant formulae due to their laxative effect. Their consumption increases fecal bolus and the frequency of depositions, reducing instances of constipation, considered one of the growing problems associated with inadequate fiber diet consumption in the modern society and neonates (Sabater-Molina et al. 2009). There are sufficient experimental data to support the hypothesis that prebiotic mixture substantially contributes to the improvement of infant formulae. Acute diarrhea is a major cause of child morbidity, for which hypotonic oral rehydration solution (ORS) is a proven therapy. However, the conventional ORS is not capable of reducing the duration and severity of the acute disease. Passariello et al. (2011) evaluated the efficacy of zinc and prebiotics (FOS and XOS) fortified ORS for treatment of diarrhea in children. The result of the randomized controlled trial showed that the zinc and the prebiotics limit diarrhea duration in patients by stimulating water and electrolyte absorption across gut mucosa and inhibiting the pathogens, respectively. The therapeutic efficacy was attributed to the synergistic relation between the additives.

### Anticancer agent and immunopotentiator

Consumption of arabinoxylan-oligosaccharides (AXOS)-enriched diet has been reported to reduce the occurrence of preneoplastic lesions in the colon of rats treated with the carcinogen 1, 2-dimethylhydrazine. So, chemoprevention potential of AXOS towards colon carcinogenesis needs to be investigated further for possible human use (Femia et al. 2010). Cellobiose 2-epimerase from *Ruminococcus albus* effectively converts lactose to epilactose. Dietary supplementation with epilactose increase cecal contents and decrease its pH, enhance lactobacilli, and bifidobacteria population, suppress clostridia or bacteroides in Wistar-ST rats. The prebiotic epilactose inhibit the conversion of primary bile acids to secondary bile acids, the promoters of colon cancer (Watanabe et al. 2008). The SCFA obtained from fermentation of GOS are known to stimulate apoptosis. Propionate has been shown to exert anti-inflammatory effect with respect to colon cancer cells (Nurmi et al. 2005). Butyrate has been evidenced to suppress expression of transcription factor NF-κB in HT-29 cell lines, whereas acetate is known to increase the peripheral blood antibody production and NK activity in cancer patients (Macfarlane et al. 2008). Vos et al. (2010) studied the the immune-modulatory effect of specific prebiotic oligosaccharides viz*.* GOS, FOS and pectin-derived acidic oligosaccharides. The supplementation exerted immunemodulatory effect during the early phase of a murine immune response. Prebiotics may reduce the incidence of degenerative diseases, such as neoplasias, diabetes, coronary diseases and infections. They also seem to promote a positive modulation of the immune system (Delgado et al. 2011). Stam et al. (2011) conducted a RCT on the effect of a prebiotic mixture supplementation in formula food on the antibody responses to Influenza and tetanus vaccination in infants during the first year of life. It was hypothesized that a prebiotic mixture of short-chain GOS, long-chain FOS and pectin-derived acidic oligosaccharides, resembling the composition of oligosaccharides in human milk, promote T Helper 1 (Th1) and regulatory T cell (Treg)-dependent immune responses and induce down regulation of IgE-mediated allergic responses. Additionally, the prebiotic administration does not interfere with the desired vaccine-specific serum antibody responses in healthy term infants (Stam et al. 2011).

### Cholesterol removal, reduction of cardiovascular disease and prevention of obesity

Synbiotic treatment of *P. acidilactici* LAB 5 with sorbitol for 1 month lowers the plasma cholesterol level of Swiss albino mice (Mandal et al. 2009). Resistant carbohydrate-rich whole grains reduce the risk of coronary heart disease (CHD). The prebiotic whole-grain intake consistently has been associated with reduced risk of the vascular instances (Harris and Kris-Etherton 2010). The concurrent increase in free ferulic acid from the enzyme-treated prebiotic durum wheat results in higher plasma ferulic acid concentration which is suggested to be potent reason for the health benefits reported for dietary fiber in cardiovascular diseases (Napolitano et al. 2009). Fermentable dietary fibers as short-chain FOS can be supplemented in foods to induce satiety and thus prevent obesity (Hess et al. 2011). Wong et al. (2010) studied the effect of soy food diet enriched with prebiotic in hyperlipidemic adults. Results showed that intake of soy-fortified prebiotic resulted in greater reductions in low-density lipoprotein cholesterol and increase of high-density lipoprotein. Co-ingestion of a prebiotic may be potentiating the effectiveness of soy foods in improving the serum cholesterol profile. Cani et al. (2009) conducted a randomized controlled trial for 2 weeks to examine the effects of prebiotic supplementation on satiety taking ten healthy human volunteers as subjects. It is concluded that prebiotic treatment and resultant lowering in hunger is linked to increased postprandial plasma gut peptide concentration. Prebiotics may prove useful tool for controlling food intake and glucose homeostasis, lowering obesity risk.

### Restoration of vaginal ecosystem

In post-menopausal women, anaerobic pathogens tend to dominate the vaginal microbiota. Prebiotics along with probiotic strains have abilities to restore vaginal ecosystem. Pliszczak et al. (2011) designed a vaginal bioadhesive delivery system based on pectinate-hyaluronic acid microparticles for probiotics and prebiotics encapsulation.

### Bacteriocin production

Prebiotic sorbitol has a positive influence on bacteriocin production from *Pediococcus acidilactici* LAB 5 isolated from vacuum-packed fermented meat product (Mandal et al. 2009). Vamanu and Vamanu (2010) studied the effect of prebiotics viz*.* inulin from chicory and dahlia, raffinose and lactulose on the synthesis of bacteriocins from *Lactobacillus paracasei* CMGB16 strain. The inhibition of *E. coli* as determined by agar well diffusion method confirmed bacteriocin production.

### In poultry, fishery, pig, cattle feed

Eco-friendly alternatives to the therapeutic use of antimicrobials are always being investigated. *Salmonellatyphimurium* is a major etiological agent of food-borne illness in humans and *Salmonella enteritidis* infection in commercial poultry is a world-wide problem. Treatment of MQ-NCSU chicken macrophage cell line with prebiotic β-1, 4-mannobiose (MNB) dose-dependently increased both phagocytic activity and *Salmonella*-killing activity of macrophages in vivo in chickens. Gene expression analysis of MNB-treated macrophages revealed significant increases in the expression of genes critical for host defense and antimicrobial activity. These data confirm that MNB possesses potent innate immune-modulating activities and can up-regulate antibacterial defenses in chicken macrophages (Ibuki et al. 2011). Prebiotic xylooligosaccharides enhanced the growth performance and digestive enzyme activities of the allogynogenetic crucian carp, *Carassius auratus gibelio* (Xu et al. 2009). The application of probiotics and prebiotics has great applications in salmonid aquaculture, resulting in elevated health status, improved disease resistance, growth performance, body composition, reduced malformations and improved gut morphology and microbial balance. However, a scientific perspective is required for understanding the mucosa–bacteria interactions to achieve optimal utilization (Merrifield et al. 2010). As consumer concern about antibiotic resistance has increased, interest in alternative supplements has grown. Ahmdifar et al. (2011) investigated the effects of different dietary prebiotic inulin levels on hematologic and biochemical parameters and some blood serum enzymes in juvenile great sturgeon (*Huso huso*). The results showed that with the increase in supplementation level of inulin, enzymes and white blood cell count increased significantly. Holstein heifer calves, when given prebiotic supplement, showed more *Lactobacilli* in their feces (Heinrichs et al. 2009). Brown *Ascophyllum nodosum* algae showed prebiotic potential in weaned piglet feed material for improving the gut flora, being an important index of the gastro-intestinal health status (Dierick et al. 2010). Oral administration of prebiotic chicory root for 16 days to pigs increased the mRNA and protein expression of Cytochrome P450 1A2 and 2A, the enzymes having crucial role in metabolism of drugs and endogenous compounds (Rasmussen et al. 2011). Dairy cattle develop hemorrhage syndrome after consuming mycotoxigenic fungi-contaminated feed. The prebiotic, Celmanax^™^ formulated with a non-living yeast cell walls or MOS, acts as an anti-adhesive for Shiga toxin producing *E. coli* O157:H7 colonization and a mycotoxin in vitro. The Celmanax^™^ also improves milk production and feed conversion efficiencies in dairy cattle (Baines et al. 2011).

### Prebiotic production from food industry wastes

Soybean whey, a by-product of tofu manufacturing, which is normally discarded, contains NDOs. Acidic fermentation of NDOs in the caecum leads to increase in mineral absorption, especially that of calcium and magnesium. In view of its potential health-promoting properties, soybean whey may be used as a valuable ingredient in functional foods (Tenorio et al. 2010). Prebiotic oligosaccharides extracted from soy sauce lees (SSLO) have growth promoting effect on *L. bulgaricus* and *S. thermophilus* (Yang et al. 2011a). Prebiotic oligosaccharides are obtained when water extractable polysaccharides (WEPs) isolated from Bengal gram husk and wheat bran are subjected to driselase enzyme hydrolysis (Madhukumar and Muralikrishna 2010). Spent osmotic sugar solution (SOS) obtained after the osmotic dehydration of carrot cubes are transfructosylated to produce prebiotic FOS (Aachary and Prapulla 2009). The solid wastes accumulated in malting industries viz*.* barley husks, spent grains and grain fragments, when processed by hydrothermal techniques, the liquor contained xylooligosaccharides. The refined oligosaccharides on fermentation generated succinate, lactate, formate, acetate, propionate and butyrate, exhibiting prebiotic potential (Gullón et al. 2011a). Connolly et al. (2010) studied the prebiotic potential of a konjac glucomannan hydrolysate. Wang et al. (2010) studied that mung bean may enhance the growth of *L. paracasei*. Gullón et al. (2011b) investigated the prebiotic potential of pectic oligosaccharide-rich refined product from apple pomace, processed by simultaneous saccharification and solid-state fermentation.

### Prebiotics from new novel sources

Yacon (*Smallanthus sonchifolius*) root is a traditional food among the Andean population in South America. It has a myriad of health-promoting properties including prebiotic effects. Though European Union is skeptical about the safety of yacon, it has a well-documented and unambiguous history of safe use and it is free of toxic substances or antinutrients (Ojansivu et al. 2011). Huang et al. (2012) investigated the effect of diosgenin, a steroid sapogenin compound from yam, on the growth of enteric LAB. Oral administration of diosgenin on murine model restored the density of fecal LAB associated with food allergic reactions. The results indicate that the steroidal sapogenins may be a novel class of prebiotics. Lupin kernel fiber, a novel legume-derived food ingredient is a prebiotic, modulating the colonic microbiota in humans, evident from the significantly higher levels of *Bifidobacterium* spp. and lower levels of the clostridia viz*. C. ramosum*, *C. spiroforme,* and *C. cocleatum* (Smith et al. 2006). Molan et al. (2009a) reported the prebiotic effect of blueberry in terms of the increase in *L.rhamnosus* and *Bifidobacteriumbreve.* It is hypothesized that the aqueous extract of blueberry could modify the bacterial profile by promoting the growth of beneficial bacteria and thereby improving gut health. Mandalari et al. (2007) studied the prebiotic effect of enzymatically derived pectic oligosaccharide-rich extract from bergamot peel and reported that bBifidobacteria and lactobacilli responded positively to the addition of the extract. Prebiotic inulin-type fructans have been isolated from the roots of traditional Chinese medicine *Morinda officinalis* or Indian mulberry (Yang et al. 2011b). The oligosaccharides of white and red-flesh pitayas (dragon fruit) have been found capable of stimulating the growth of lactobacilli and bifidobacteria (Wichienchot et al. 2010). The β-glucans extracted from *Pleurotus* sp. (pleuran) mushrooms have been used as food supplements due to their immunosuppressive activity and stimulation of probiotics. This exploitation of fruit body extracts extends the use of mushrooms *P. ostreatus* and *P. eryngii* for human health (Synytsya et al. 2009). Glycated pea proteins enhanced the growth of gut commensal bacteria, particularly lactobacilli and bifidobacteria, because the energy contained in glycated pea proteins, partially inaccessible for gastric enzymes, could be salvaged by gut microbiota. Such changes in microbial composition may beneficially impact the intestinal environment and exert a health-promoting effect in humans (Dominika et al. 2011). Molan et al. (2009b) studied the effect of selenium-containing green tea (SGT) and China green tea (CGT) on the in vitro growth of lactobacilli and bifidobacteria. SGT showed significantly higher phenolic contents, higher reducing activity, higher DPPH free-radical scavenging activity and higher ferrous-ion chelating activity than CGT. The addition of aqueous extracts of SGT to Mann-Rogosa-Sharpe (MRS) broth resulted in significant increase in the numbers of *L. rhamnosus* and *B. breve*. The higher prebiotic activity of SGT over CGT may be related to the higher phenolic contents and minerals, notably selenium. The prebiotic health potential of the polysaccharides from seaweeds is increasingly being studied in keeping with the increased consumption of whole seaweeds or the purified polysaccharides (Gupta and Abu-Ghannam 2011). The sulfated heteropolysaccharide, ulvan, extracted from green algae *Ulva rigida* is resistant to both human digestive tract enzymes and degradation by colonic bacteria. So, it is expected that this polysaccharide can be deemed as a prebiotic and further hydrolyzed to functional oligosaccharides (O’Sullivan et al. 2010). Wang et al. (2006) studied that alginate oligosaccharide-rich diet stimulated the growth of *Bifidobacterium bifidum* ATCC 29521 and *B. longum* SMU 27001, decreasing the abundance of enterobacteriaceae and enterococci. Ramnani et al. (2011) studied that low-molecular-weight polysaccharides derived from agar and alginate of seaweed *Gelidium* CC2253 showed a significant increase in bifidobactera population. This seaweed easily fermented into short-chain fatty acid by gut bacteria and exhibits potential to be used a novel source of prebiotics. Fructans from agave (*Agave tequilana* Weber var. azul) are designed to produce a mixture of functional prebiotic FOS and sweetening powder (Ávila-Fernández et al. 2011). Neoagaro-oligosaccharides (NAOS), obtained from enzymatic hydrolysis of agarose, are found to be highly resistant to enzymes of the upper gastrointestinal tract, which significantly stimulated the growth of bifidobacteria and lactobacilli. These results indicated that NAOS possessed great prebiotic effect, which could be beneficial to the host (Hu et al. 2006). Vidanarachchi et al. (2009) isolated water-soluble prebiotic compounds from Australian and New Zealand plants *Arthropodium cirratum* and *Cordyline australis*. Inulin extracted from edible burdock also showed prebiotic properties (Li et al. 2008).

### Prebiotic supplementation in food

Cynobacteria *Spirulina* sp. has huge nutritional value being a rich source of amino acids, high proteins, calcium, vitamins A, B_2_, B_12_, E, H, K, essential minerals, iron, ω-6 fatty acids and trace elements. The stimulatory effect of aqueous suspensions of *Spirulina platensis* dry biomass has been reported on four lactic acid bacteria in milk. The addition of dry *S. platensis* to milk (6 mg/ml) stimulated the growth of *Lactococcus lactis*, to a remarkable 27% (de Caire et al. 2000). Nowadays, when the dairy industry is supplementing milk with minerals, vitamins and antioxidants, it would be of interest to consider the possibility of adding Spirulina biomass to fermented milk to induce a faster production of lactic acid bacteria in the dairy product as well as in the gut. Dietary fibers exhibiting high viscoelasticity imparts breads with better sensory perception, lower digestible starch and higher resistant starch contents, lowering down the in vitro expected glycemic index (Angioloni and Collar 2011). Damen et al. (2012) investigated the in situ production of prebiotic AXOS during bread making. Xylanase from fungi *Hypocrea jecorina* cleaved the arabinoxylan fraction of the cereal resulting in AXOS content of 2.1%. Rye or wheat bran fortification in the AXOS-rich dough further enhanced the bread quality. The prebiotic effect of the fermented cashew apple juice, containing oligosaccharides, is evaluated through the *Lactobacillus johnsonii* B-2178 growth (Vergara et al. 2010). Long-chain and short-chain inulin, when combined in different proportions and added (7.5 g/100 g) to low-fat custards demonstrate improved rheological and sensory properties. The use of this blend combined with carrageenan gives thicker, creamier custard which finds consumer preference over the full-fat custard (Tárrega et al. 2010). The addition of prebiotic lactulose in skim milk increased the probiotic counts (particularly *B. lactis)*, the acidification rate, amount of lactic acid acidity, concurrently reducing the fermentation time (Oliveira et al. 2010). Functional *petit*-*suisse* cheese, made with the combination of different prebiotics and probiotics resulted in promising health benefits (Cardarelli et al. 2007).

The diversification of food products and the growing interest in health life requires innovations from food industry. Cholesterol-lowering dietary fiber, oat β-glucan and the prebiotic inulin may be added to bread to influence their rheological properties, bread quality and crumb microstructure. On screening the microorganisms in the milk whey culture, *Propionibacterium freudenreichii* ET-3 was found to stimulate the growth of bifidobacteria in the colon, but not the growth of pathogens. The active substance was identified as 1, 4-dihydroxy-2-naphthoic acid (DHNA). In healthy volunteers, the ingestion of milk whey culture significantly increased the population of bifidobacteria in total fecal bacterium. In the TNBS-induced colitis model of rats, milk whey culture significantly accelerated the healing of the colitis in a dose-dependent manner. It has been reported that DHNA inhibited the lymphocyte infiltration through reduction of MAdCAM-1 in DSS colitis model of mice and that the ingestion of milk whey culture was effective in the treatment of ulcerative colitis in human pilot study. The findings of Uchida et al. (2007) suggest that milk whey culture is a useful prebiotic for the therapy of inflammatory bowel disease. Ice creams supplemented with *L. casei* and 2.5% inulin showed good nutritional and sensory properties (Criscio et al. 2010). Rößle et al. (2010) developed potentially synbiotic fresh-cut apple wedges by applying probiotic bacteria (*L. rhamnosus* GG) and prebiotics oligofructose and inulin. Fructan analysis showed that all prebiotics remained stable over the 14-day storage period and an intake of 100 g of apple supplied 2–3 g of prebiotics. Different dehydrated prebiotic fibers viz*.* oat bran, β-glucan and green banana flour provided substratum for adherence and trehalose acted as a cell protectant prolonging the viability of *L. casei*. In the sensory evaluation, the prebiotic oat bran added to a dairy fruit beverage has been well accepted by consumers (Guergoletto et al. 2010). Apple purees enriched with two commercial FOS prebiotics (Beneo GR^®^ (inulin) and HSI^®^) showed stability for 30 days’ storage at 4 °C (Keenan et al. 2011). However, ample quantities of prebiotics were required to deliver prebiotic effect and high hydrostatic pressure posed the risk of certain prebiotics hydrolysis. Buckwheat diet showed an increase in aerobic mesophilic and lactic acid bacteria content compared with control. *Lactobacillus plantarum, Bifidobacterium* spp. and *B. lactis* were found in buckwheat diet, confirming its candidature as a prebiotic product (Préstamo et al. 2003). Buckwheat diet also decreased the level of cholesterols in serum by enhancing their removal through feces, improved diabetes by inhibiting the absorption of sugars and enhanced growth of beneficial intestinal bacteria. *d*-*chiro*-Inositol, found in buckwheat contributed to the improvement of insulin resistance by potentiating the action of insulin (Takahama et al. 2011). Lyophilized inulin syrup made from Jerusalem artichoke tuber was used as a prebiotic ingredient in the small-scale manufacture of wafer crackers. Sensory analysis revealed a significant influence of product formulation on appearance, flavor and texture of the crackers (Hempel et al. 2006). The impact of incorporation of prebiotic arabinoxylan oligosaccharides (AXOS) on the quality of a sugar-snap cookie type was investigated. Replacing up to 30% of the initial sucrose level by AXOS resulted in cookies with comparable size, but of darker color than the control cookies. Pareyt et al. (2011) proposed the possible role of AXOS as sucrose substitute, which has practical implications from a health point of view, a reasonable consumption of which may exert beneficial biological effects. Peach-flavored yogurts containing the prebiotic did not significantly influence the flavor and so did not much impact consumer acceptance (Gonzalez et al. 2011). Allgeyer et al. (2010) studied the popularity of dairy products fortified with prebiotics and probiotics, motivated with the consumer desire for good flavored healthy foods. Sensory profile of drinkable yogurts made with prebiotics viz*.* soluble corn fiber, polydextrose and chicory inulin were assessed. Polydextrose treatment seemed to be an acceptable vehicle to deliver the probiotic health effects at the end of the 30-day storage period. Mitsou et al. (2010) evaluated the in vivo prebiotic potential of barley β-glucan and concluded that it induced a strong bifidogenic effect. It is reported that daily intake of a cake containing barley β-glucan is well tolerated and demonstrated significant bifidogenic properties in older healthy volunteers.

### Prebiotic production by various techniques and enhancement of prebiotic potential

**Fig. 2:**
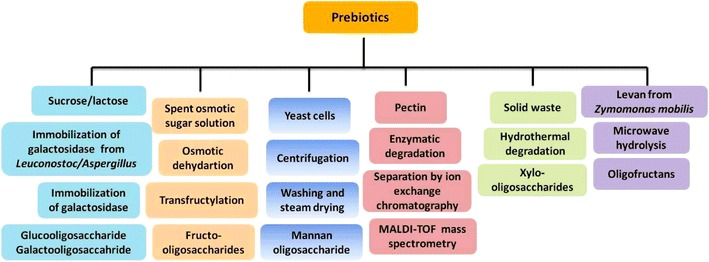
Various methods of prebiotics production

Currently, the technology for the production of oligosaccharides is limited to extraction from plant sources, acid or enzymatic hydrolysis of polysaccharides or synthesis by transglycosylation reactions. Prebiotic oligosaccharides may also be produced using Leuconostoc fermentation and restricting the polymer size by addition of maltose or galctose. Sugar beet pectin was degraded enzymatically and separated by ion exchange chromatography into series of highly purified homogalacturonide and rhamnogalacturonide oligosaccharides. MALDI-TOF/TOF mass-spectrometry was used to determine the size and structural features. In vitro microbial fermentation by human fecal samples showed a different response to the DP4 and DP5 homogalacturonides on the ratio between *Bacteroidetes* and *Firmicutes*. This indicated that pectic oligosaccharides with only slightly different structures have significantly different biological effects (Holck et al. 2011). β-galactosidase from *Aspergillus oryzae* was immobilized by different methods for the synthesis of GOS from lactose at high concentrations. At optimal conditions, the conversion was reported as 30%. In the sequential batch production, 8,500 g of GOS per gram of enzyme preparation was produced after ten batches, the yield of which was further expected to increase by biocatalyst replacement (Huerta et al. 2011). A recombinant *α*-glucosidase from *Thermoanaerobacter ethanolicus* JW200, cloned and expressed in *E. coli* showed strong transglycosylation activity in presence of maltose. The transglucosylation products were identified to be prebiotic isomaltooligosaccharides (Wang et al. 2009). A commercially available endo-inulinase from *Aspergillus niger* was successfully immobilized onto a chitin carrier for production of FOS from inulin (Quang et al. 2011). A fructofuranosidase enzyme extracted from *Xanthophyllomyces dendrorhous* 269 exhibited a high transfructosylation activity, and it has potential for the industrial production of prebiotic neo-FOSs (Chen et al. 2011). The α-glucosidase from *Bacillus licheniformis* TH4-2 was used in the glucosyl transfer reaction for the synthesis of a trisaccharide oligosaccharide. The prebiotic nature of this product was suggested from its hydrolysis resistance to enzymes of rat intestine (Nimpiboon et al. 2011). Levan, a polysaccharide from *Zymomonas mobilis*, was hydrolyzed in a microwave oven to obtain oligofructans that beneficially affect the host by selective stimulation of probiotic bacteria in the colon (de Paula et al. 2008). Yeast cell wall was ruptured by centrifugation and separated from the yeast extract, washed, dried and pasteurized on a steam drum dryer to harvest the prebiotic mannan-oligosaccharides. The various methods of prebiotics production have been illustrated in Fig. 2.

Vaidya and Sheth (2010) studied the resistant starch content in raw and processed cereals and their products. Roasting, baking and boiling increased the resistant starch content followed by shallow frying, whereas steaming and frying decreased the content. The puffed, flaked and extruded cereal products obtained from market when analyzed also showed very less retention of resistant starch content. Storage of different cereal products at 4 °C up to 12 and 24 h significantly increased resistant starch content.

### Future perspectives of prebiotic research

Recent research findings have shown hitherto unknown, health potentials of prebiotics. Everard et al. (2011) investigated that in obese mice, prebiotic feeding decreased firmicutes abundance, with simultaneous improvement in glucose tolerance, reduced fat accumulation, oxidative stress, and inflammation. It was concluded that prebiotics induced gut microbiota modulation, improves glucose homeostasis and it may be a key strategy in diabetes therapy. Grüber et al. (2010) assessed the effect of supplementation of an infant formula with prebiotic on the occurrence of atopic dermatitis. The immunoactive oligosaccharides containing prebiotic could effectively prevent of atopic dermatitis in low atopy risk infants. Till now, prebiotic concept has been confined to nutraceutical and pharmaceutical domain. But, it is making rapid strides towards cosmeceutical spheres also. It is hypothesized that prebiotic substances may be applied to modulate any microbial community to achieve advantageous effects. Prebiotics are expected to cope with skin issues as inflammation and smell. Prebiotic products have shown a significant 91% success in human trial study (Bockmühl et al. 2007), thus proving they can effectively decrease *Propionibacterium acnes* population and treat the acne.

## Conclusion

The introduction of functional compounds like prebiotics in the diet seems to be an attractive alternative to ameliorate the quality of life ridden with obesity, cancer, hypersensitivity, vascular diseases and degenerative ailments. The enormous functional metagenomic data provided by the Human Microbiome Project is expected to revolutionize the prebiotic research by rational production of desired prebiotic molecules with specific functional properties. There are claims that prebiotics are capable of preventing weight gain in adolescents and improving immunity in geriatrics and infants. Prebiotics are expected to enter the dermatological sector and boost skin health. Also, it is being mooted that prebiotics will eventually replace the antibiotics used as growth stimulants in apiary, fishery, poultry and animal husbandry. The clinical significance of the prebiotics remains to be clarified, the claims of efficacy proved and underlying mechanism decoded. Owing to its wide range of preventative and therapeutic possibilities prebiotics research is certainly catching momentum.
